# CARE COMPARE STAR RATINGS AND FAMILY SATISFACTION IN MARYLAND NURSING FACILITIES

**DOI:** 10.1093/geroni/igad104.3208

**Published:** 2023-12-21

**Authors:** Roberto Millar, Christin Diehl, Nancy Kusmaul, Ian Stockwell

**Affiliations:** The Hilltop Institute at UMBC, Baltimore, Maryland, United States; The Hilltop Institute at UMBC, Baltimore, Maryland, United States; The Hilltop Institute at UMBC, Baltimore, Maryland, United States; University of Maryland Baltimore County, Baltimore, Maryland, United States

## Abstract

Nursing facilities provide critical services and supports to individuals with long-term care needs. The quality of care in nursing facilities varies depending on facility structural characteristics. Moreover, the measurement and perceptions of what constitutes quality of care varies across stakeholders. We used publicly available data to examine the association between family satisfaction with care and the Centers for Medicare & Medicaid Services’(CMS’s) Care Compare five-star quality ratings in the context of facility characteristics. Facility-level data of family satisfaction with care were merged with CMS’s five-star star ratings of 220 Maryland nursing facilities in 2021. Using univariate and bivariate statistics, we explored differences in family ratings and five-star ratings across facility ownership (for-profit vs. non-profit), geographic location (urban vs. rural), and average resident occupancy (1-60, 61-120, and 121+). Relationships were examined across overall ratings, as well as across subdomain of the two quality rating frameworks (e.g., staffing, autonomy, health inspections). Family members of residents in non-profit, rural, and low-occupancy facilities rated facilities higher. Non-profit and low-occupancy facilities were statistically more likely to be rated four or five stars, while no significant association was observed across geographic location. The association between subdomain-specific family satisfaction and star ratings varied across facilities of different structures. Findings emphasize the need for comprehensive quality of care frameworks that consider views of quality across stakeholders and types of facilities. A clear understanding of nursing facility structure and quality of care is critical to advance data-driven decision making.

